# Sustained Transmission of Pertussis in Vaccinated, 1–5-Year-Old Children in a Preschool, Florida, USA

**DOI:** 10.3201/eid2202.150325

**Published:** 2016-02

**Authors:** James Matthias, P. Scott Pritchard, Stacey W. Martin, Cristina Dusek, Erika Cathey, Rebecca D’Alessio, Marjorie Kirsch

**Affiliations:** Florida Department of Health, Tallahassee, Florida, USA (J. Matthias, P.S. Pritchard, C. Dusek, E. Cathey);; Centers for Disease Control and Prevention, Atlanta, Georgia, USA (S.W. Martin);; Florida Department of Health in Leon County, Tallahassee (R. D’Alessio, M. Kirsch)

**Keywords:** pertussis, Bordetella pertussis, bacteria, outbreak, sustained transmission, preschool, preschool children, vaccination, vaccines, respiratory infections, Florida

## Abstract

In September 2013, local county health officials in Tallahassee, Florida, USA, were notified of a laboratory-confirmed pertussis case in a 1-year-old preschool attendee. During a 5-month period, 26 (22%) students 1–5 years of age, 2 staff from the same preschool, and 11 family members met the national case definition for pertussis. Four persons during this outbreak were hospitalized for clinical management of pertussis symptoms. Only 5 students, including 2 students with pertussis, had not received the complete series of vaccinations for pertussis. Attack rates in 1 classroom for all students who received the complete series of vaccinations for pertussis approached 50%. This outbreak raises concerns about vaccine effectiveness in this preschool age group and reinforces the idea that recent pertussis vaccination should not dissuade physicians from diagnosing, testing, or treating persons with compatible illness for pertussis.

## Abstract 2

Monitoring of vaccine performance is necessary to identify outbreaks or emerging epidemiologic trends.

In the United States, incidence of pertussis is greatest among infants, children 7–10 years of age, and adolescents (*1*). During 2000–2012, reported pertussis cases increased >6-fold from 7,867 cases to 48,277 cases (*2*). One potential contributing factor for increased incidence of pertussis is waning immunity after acellular pertussis vaccination (*3*,*4*).

In September 2013, the Florida Department of Health in Leon County (DOH–Leon, Tallahassee, FL, USA) was notified of a PCR result positive for *Bordetella pertussis* for a 1-year-old vaccine-exempt preschool student. Treatment, chemoprophylaxis, and pertussis education were provided to household close contacts, classmates, parents of classmates, and staff associated with the classroom for this student. The investigation identified a 3-year-old sibling who had illness clinically compatible with pertussis before onset of pertussis in the 1-year-old student. This sibling did not attend the preschool.

On December 11, DOH–Leon received a report that a 1-month-old infant had a PCR result positive for pertussis. A public health investigation determined that the vaccinated 3-year-old sibling and mother of this infant had illnesses clinically compatible with pertussis and disease onset before that of the infant. The sibling attended the same preschool as the initial 1-year-old student, and the mother was a substitute teacher at the preschool. Less than 1 week later, another 3-year-old child who attended the preschool showed a PCR result positive for pertussis.

In response to these reports, an outbreak investigation was initiated. On December 19, the local DOH staff conducted a site visit to the preschool to prevent further transmission and determine the incidence of pertussis among students, household close contacts, and staff.

## Methods

The preschool had 117 students 10 months–6 years of age and 26 staff. On December 19, the preschool director and staff were queried about any additional students or staff members with a cough illness. At this time, the preschool instituted school wide cough illness surveillance and reported any new cough illnesses to DOH. For newly identified case-patients and their contacts, treatment and prophylaxis were administered according to guidelines from the Centers for Disease Control and Prevention (CDC; Atlanta, GA, USA). All symptomatic persons, or their parents if they were <18 years of age, were interviewed by using a standardized case report form to record onset dates, demographics, symptoms, healthcare visits, laboratory testing, treatment, and vaccination status. Pertussis vaccination status for case-patients and all students was cross-referenced in the Florida immunization registry and with preschool vaccination records.

The Council of State and Territorial Epidemiologists (CSTE) 2013 case definition for pertussis was used to classify cases, with the exception that serologic analysis performed at CDC was considered a confirmatory laboratory test (*5*). In addition, persons with only school-based epidemiologic links to a laboratory-confirmed case were classified as probable cases, rather than confirmed cases. All other epidemiologic links, such as household links, were classified as confirmed per CSTE case definition. One residual nasopharyngeal specimen that showed a PCR result positive for pertussis at a commercial laboratory and 3 serum samples collected retrospectively from consenting case-patients (adult) were forwarded to CDC for confirmation of *B. pertussis* infection.

On January 7–8, 2014, DOH staff administered an onsite cough illness questionnaire to student and staff households (completion rate 98%). The questionnaire sought to capture any cough illness, classic symptoms of pertussis, and duration of illness since December 1, 2013. All but 3 student households and 1 staff member household completed the cough illness questionnaire. Case data were analyzed for several factors, including age, classroom, number of vaccinations, duration from most recent vaccination to symptom onset, and case classification status.

Vaccine effectiveness was calculated as (1 – relative risk) × 100 for the cohort of children attending the preschool (*6*). Relative risk was defined as the attack rate (AR) in fully vaccinated children divided by the AR in children whose vaccination status was not up to date. All children attending the preschool were age-eligible to have received >3 doses of pertussis vaccine (DTaP). Children <18 months of age were considered fully vaccinated if they had received 3 doses of DTaP. All other children were considered fully vaccinated if they had received >4 doses of DTaP.

## Results

Eleven cases were detected during September 2013–January 2014 and classified as confirmed: 5 laboratory confirmed (3 by PCR specific for IS*481*; 1 by PCR specific for IS*481*, HIS*1001*, PIS*1001I*, *ptxS1*, and RNaseP; and 1 by serologic testing at CDC) and 6 epidemiologically linked household contacts (Figure). Twenty-eight cases were classified as probable (total of 39 confirmed and probable cases). Twenty-six students 1–5 years of age (AR 22%) and 2 staff (AR 7%) were identified as having pertussis (Table). The remaining 11 case-patients were linked to the preschool: 9 were household contacts and 2 were camp counselors who had contact with a sibling of a laboratory-confirmed case-patient who attended the preschool.

**Figure:**
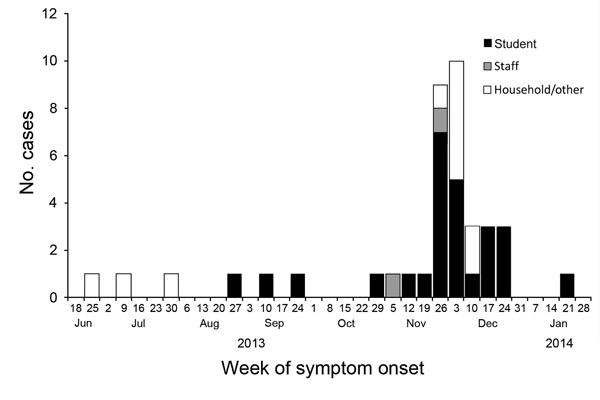
Epidemic curve of confirmed and probable pertussis cases during an outbreak in a preschool, by week of symptom onset, Florida, USA, 2013–2014. A total of 26 students (black bars), 2 staff (gray bars), and 11 household/other epidemiologically linked persons (white bars) were involved in this outbreak.

**Table:** Characteristics of students, staff, and household members associated with an outbreak of pertussis in a preschool, Florida, USA*

Characteristic	Case classification status				Characteristic comparisons	
	Confirmed	Probable	Noncase		% Cases, n = 39	Attack rate, %
Link to preschool						
Student	4	22	91		67	22
Staff	2	0	26		5	7
Household/other†	5	6	ND		28	ND
Hospitalized	2	2	ND		10	ND
Symptoms						
Apnea	2	2	ND		10	ND
Cough >2 wk	11	28	ND		100	ND
Inspiratory whoop	5	2	ND		18	ND
Paroxysmal cough	9	13	ND		56	ND
Posttussive vomiting	4	4	ND		21	ND
Age‡						
<18 mo	1	1	7		8	22
18 mo–4 y	2	13	41		58	27
≥4 y	1	8	43		35	17
Up-to-date pertussis vaccinations‡	3	21	88		92	21
No. pertussis vaccinations‡						
<3	1	1	3		8	40
3	0	1	4		4	20
>4	3	20	81		88	22
Time from vaccination to symptom onset, y‡§						
<1	1	6	39		27	15
1–<2	1	7	22		31	27
2–3	1	5	13		23	32
>3	1	4	14		19	26
Classroom‡						
1A	1	1	11		8	15
2A	0	1	11		4	8
2B	0	3	10		12	23
3A	0	5	12		19	29
3B	2	6	9		31	47
4A	1	1	7		8	22
4B	0	1	9		4	10
4C	0	2	7		8	22
4D	0	2	6		8	25
Kindergarten	0	0	9		0	0

*ND, not determined. †Other, symptomatic camp counselors unaffiliated with the preschool who had contact with children or siblings that attended the preschool. ‡Values and calculations are only for students in the preschool. §For noncases, time from vaccination to symptom onset used January 1, 2014, as onset date.

Four case-patients 1 month–2 years of age, including 2 students, were hospitalized. Only 1 of the hospitalized case-patients had received >3 doses of pertussis vaccine (2 children were too young for 3 doses and 1 child who had received 1 dose was on a delayed schedule). Lengths of hospitalization ranged from 1 to 5 days, and duration of cough ranged from 14 to 50 days. Three of the four hospitalized case-patients had posttussive vomiting, 2 had paroxysmal cough, 2 had inspiratory whoop, and 1 had stridor.

All 39 case-patients had a cough illness for >2 weeks, which is consistent with the CSTE case definition (*5*). The average duration of illness for all case-patients was estimated to be 23 days, which is an underestimate because 25 case-patients were still symptomatic at time of last interview. Fourteen (54%) of 26 students and 11 (85%) of 13 case-patients who were not students had additional symptoms consistent with pertussis (Table). A paroxysmal cough (56%) was the most common additional symptom identified.

Only 5 of 117 children in the preschool had not received the complete series of vaccinations. Of these 5 children, 2 were case-patients and both had received >1 dose of pertussis vaccine: the hospitalized 1-year-old child who had received a single dose and a 3-year-old child who had received only 2 doses. The other 3 children were unvaccinated but did not have pertussis.

Of the 33 children who had pertussis, 28 had received >3 pertussis vaccinations, and 23 had received >4 vaccinations. Vaccine effectiveness among children attending the preschool was estimated to be 45.0% (95% CI −70.4% to 82.2%). The average number of days from last vaccination to onset of symptoms for the students was only 667 days (≈22 months), and 7 (27%) children had been vaccinated within the previous year.

ARs among students by classroom ranged from 0% to 47%; 6 classrooms had an AR >20% for students. The 2 classrooms with 3-year-old students had the highest ARs. Moreover, an AR of 48% was identified in 1 of these classrooms in which all 17 students had received the complete series of vaccinations. This classroom had an infectious staff member with laboratory-confirmed pertussis during the outbreak. Children 2–3 years of age were at 2.2 times (95% CI 1.0–4.9 times) greater risk for pertussis infection than children 4–6 years of age. When this analysis was restricted to only children 3 years of age, the risk increased to 2.5 times (95% CI 1.1–5.5 times).

## Discussion

This investigation highlights an outbreak of pertussis in a preschool with few vaccine exemptions. To our knowledge, sustained transmission of pertussis in a vaccinated cohort of 1–5-year-old children has not been reported in the United States. Short-duration vaccine effectiveness estimates for children receiving >3 doses of acellular pertussis vaccines have been described at >80% (*4*,*7*,*8*). Although the small number of nonvaccinated children in the preschool resulted in a vaccine effectiveness that had extremely wide CIs that overlap 0, the low estimate, coupled with documented sustained transmission over a period of months, raises concerns about inadequate protection against pertussis in an age group believed to be well protected by acellular pertussis vaccination.

Poor performance of a vaccine in a defined cohort might suggest a provider-level failure to store, use, and administer the vaccine properly. Although we did not assess vaccine storage and handling practices, children from this investigation were seen by multiple providers in the community. Moreover, no general increase in reported pertussis incidence was observed in the county at the same time as this outbreak.

Although the number of cases confirmed by laboratory testing was low (13%), many of the case-patients had substantial illness consistent with pertussis. In addition, *B. pertussis* infections were confirmed in persons from 5 households over a 5-month period. Confirmatory laboratory testing at CDC provided further evidence of a *B. pertussis* outbreak. The 3 nonvaccinated students in whom illness did not develop were not assessed for prior evidence of infection.

The use of a focused cough illness questionnaire for case ascertainment might have captured cough illnesses that met the case definition, but might not have been pertussis, during December 1, 2013–January 7, 2014. However, because the questionnaire focused on this narrow period, to minimize recall bias, additional cases of pertussis before December 1, 2013, might not have been identified.

Over the course of the outbreak, mass prophylaxis was provided only to the classroom with 1-year-old children after laboratory confirmation of the first reported case because of concerns about the risk for severe pertussis in this younger age group. Given the limited laboratory testing early during the outbreak and to be consistent with adherence to CDC guidelines, classroom-wide chemoprophylaxis was not provided for laboratory-confirmed cases in older children. Chemoprophylaxis was provided to household and high-risk contacts. As a result, effects of school-wide or classroom-wide chemoprophylaxis were not assessed. No staff were identified as being pregnant during the outbreak. However, early during the outbreak, the 1-year-old child of a rotating staff member (use of postexposure prophylaxis by this staff member was not known) was hospitalized with laboratory-confirmed pertussis in December, despite use of prophylaxis in September by the class in which the mother worked.

As part of this investigation, it was apparent that many physicians were hesitant to provide a diagnosis of pertussis and did not test for this disease, given the recent vaccination history of the patients and despite reporting of an ongoing laboratory-confirmed pertussis outbreak. The spectrum of illness for pertussis in vaccinated children can vary widely and is often mild, with few classic symptoms of pertussis (*9*). Hesitation by providers in reporting presumptive pertussis delays public health response to prevent continued transmission of pertussis in the community. Thus, recent pertussis vaccination should not preclude diagnosis, testing, and treatment of presumptive pertussis cases (*10*).

Although all children in the classroom had received the complete series of vaccinations for pertussis, the classroom with the highest AR was one in which a teacher with a laboratory-confirmed case of pertussis who had not received a Tdap booster vaccination, worked throughout her illness. Three of the students showed symptom onset before the teacher, and 5 students, including 1 laboratory-confirmed case-patient, had symptom onset 6–13 days after the teacher showed symptom onset. Improved efforts toward early diagnosis and appropriate treatment to mitigate transmission and booster vaccinations for adults in situations in which prolonged close contact between children, especially children <1 year of age, and adults occur could be considered (*11*).

Reports of genetic changes in circulating *B. pertussis* have raised concern that this organism could be adapting to vaccine-induced immunity (*12*,*13*). Given these reports and the increased levels of circulation of pertussis among older age groups with documented waning of immunity, further monitoring of acellular pertussis vaccine performance in preschool-age children is necessary to determine if this outbreak was an isolated finding or possibly identification of an emerging epidemiologic trend.
